# CO_2_ laser-assisted sclerectomy with sclerostomy for uveal effusion syndrome

**DOI:** 10.3389/fmed.2026.1775395

**Published:** 2026-04-10

**Authors:** Mingyue Luo, Ying Chen, Lu Sun, Youxin Chen, Meifen Zhang, Huan Chen, Rongping Dai

**Affiliations:** 1 Department of Ophthalmology, Peking Union Medical College Hospital, Chinese Academy of Medical Sciences, Beijing, China; 2 Beijing Key Laboratory of Fundus Diseases Intelligent Diagnosis & Drug/Device Development and Translation, Beijing, China; 3 Key Lab of Ocular Fundus Diseases, Chinese Academy of Medical Sciences, Beijing, China; 4 Xi’an People’s Hospital (Xi’an Fourth Hospital), Shannxi Eye Hospital, Xi’an, Shannxi, China

**Keywords:** choroidal effusion, CO_2_ laser-assisted sclerectomy, sclerectomy, surgery, uveal effusion syndrome

## Abstract

**Background:**

Surgical treatment of idiopathic uveal effusion syndrome (UES) is challenging with high risks. CO_2_ laser-assisted sclerectomy surgery (CLASS) is potentially a semiquantitative, quick and safe surgical option. This study aimed to describe the feasibility and early outcomes of CO_2_ laser-assisted sclerectomy surgery (CLASS) with sclerostomy for idiopathic uveal effusion syndrome (UES).

**Methods:**

This was a prospective descriptive case series. After fully exposed, the sclera was ablated with CO_2_ laser beam repeatedly until a bluish hue was visible. A crescent blade was used to make scleral incision in the same plane with the floor of the scleral pool. The lamellar sclera was cut, and the scleral pool was enlarged. A 1 × 1 mm^2^ sclerostomy was created in each sclerectomy bed with a 15 degree stab blade.

**Results:**

The technique was used in three eyes of three patients with UES and exudative retinal detachment (RD). After a mean follow-up of 410 days (range, 34–825 days), all patients demonstrated sustained improvement of RD, with complete resolution in two cases. No adverse events were observed during the extended follow-up period.

**Conclusion:**

CLASS with sclerostomy appears to be a feasible and durable technique for UES in this small series, though further studies are warranted.

## Background

Scleral-thinning procedures, which include creation of scleral flaps or windows with or without sclerostomy, are recommended in patients of severe uveal effusion syndrome (UES) who do not respond well to medications (1). These procedures help drain the uveal effusion by decreasing scleral resistance and increasing transscleral outflow (2). One of the key factors is to create a scleral pool deep enough to drain the effusion effectively, with the remaining sclera strong enough to maintain the shape of the eyeball and prevent potential complications, such as traumatic expulsive hemorrhage, scleral ectasia, etc. (3). Larger and deeper resections, although more effective theoretically, carry higher risk of these complications, imposing surgeons to pursue the delicate balance between efficacy and safety.

The control of thickness is crucial in sclerectomy, relying largely on the experience and surgical skills of surgeons (4). The carbon dioxide (CO_2_) laser, which is widely applied in CO_2_ laser-assisted sclerectomy surgery (CLASS), an improved version of nonpenetrating deep sclerectomy (NPDS), offer another option. It is highly effective in photoablation of dry tissue and coagulation of bleeding vessels, with immediately absorption of energy by fluid, rendering the safety and efficacy to create a thin percolation membrane of sclera (5). These properties make CO2 laser a perfect candidate to create the scleral pool in UES, as it ablates the sclera piecemeal in a delicate way with lower risk of complications. In addition, the technique is relatively simple and effective, requiring a shorter learning curve and lower technical demands (6, 7) compared with the traditional sclerectomy.

In this report, we describe CO_2_ laser assisted lamellar-sclerectomy with sclerostomy, which was performed in three eyes of three patients with UES as described below. This semiquantitative, quick and safe method may provide satisfactory surgical results with effective absorption of uveal effusion.

## Methods

This was a prospective descriptive case series at Peking Union Medical College Hospital from January 1, 2023 to December 1, 2023. The protocol was approved by the Peking Union Medical College Hospital Review Board (S-K631) and was conducted in accordance with the tenets of the Declaration of Helsinki. Informed consents were acquired from all patients.

## Surgical technique

All surgeries were performed by one of the authors (R.D.) under retrobulbar anesthesia with 2 mL (40 mg) lidocaine and 2 mL (20 mg) ropivacaine. Limbal 360° conjunctival peritomy was performed, followed by radial conjunctival incisions in the superior-nasal and inferotemporal quadrants. Four rectus muscles were isolated and secured with a modified muscle hook and 2–0 silk tractional sutures. Remnant tissues of Tenon’s capsule were removed until the sclera was fully exposed. CO_2_ laser beam of 4 × 2.4 mm^2^ in size and 20 watts in energy (OT-135 CO_2_ laser system, IOPtiMate; IOPtima Ltd., Ramat Gan, Israel) was applied at the equator. The ablated area was dried with cotton swabs.

**Figure 1:**
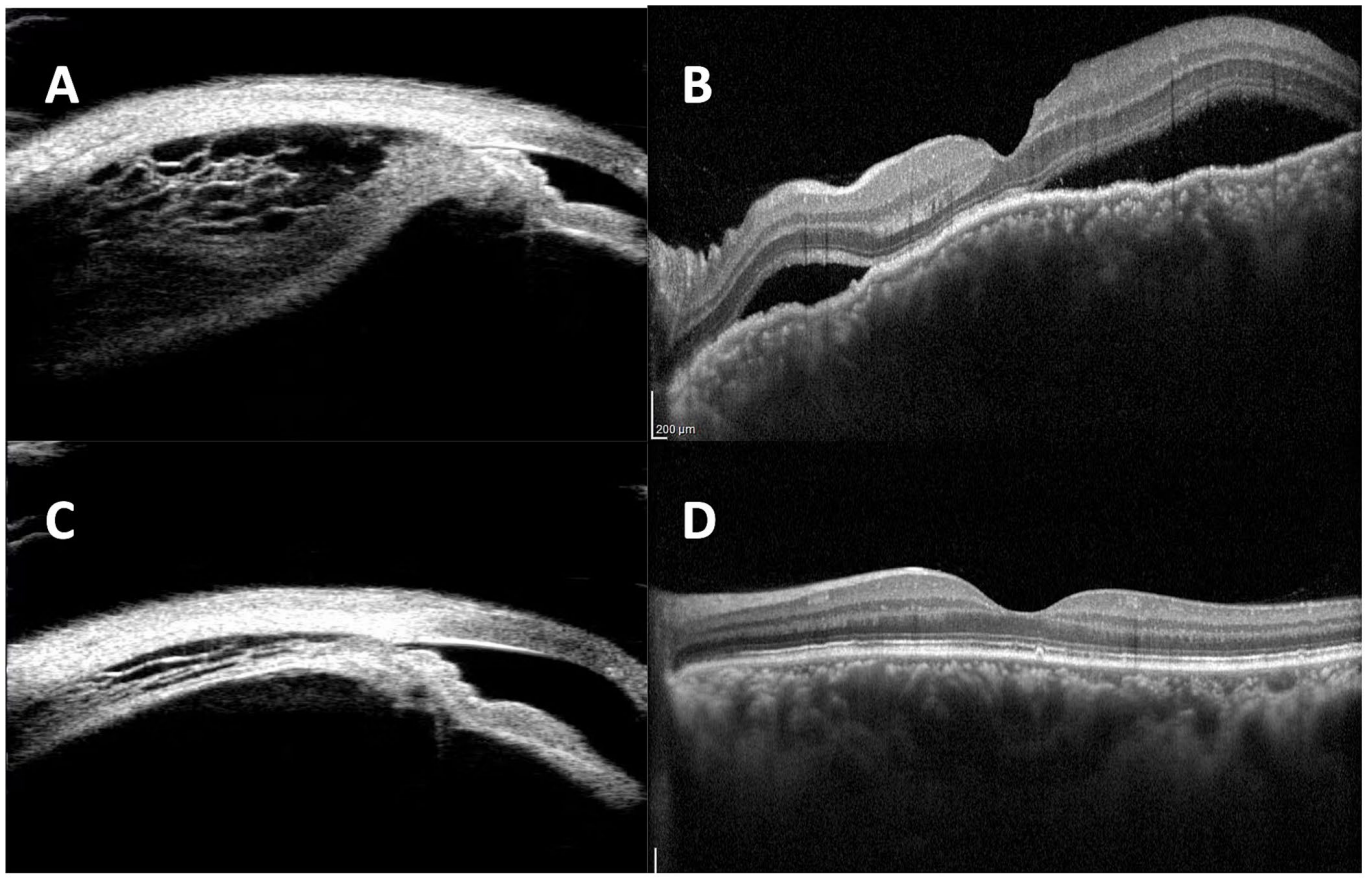
Intraoperative view of the surgical procedures. **(A)** After fully exposed, the sclera was ablated with CO_2_ laser beam repeatedly until a bluish hue was visible. **(B)** A crescent blade was used to make scleral incision in the same plane with the floor of the scleral pool. **(C)** The lamellar sclera was cut, and the scleral pool was enlarged. **(D)** A 1 × 1 mm^2^ sclerostomy was created in each sclerectomy bed with a 15° stab blade.

The procedure was repeated for 9–17 times until the bluish hue of the choroid was visible, indicating that approximately 80% of scleral thickness had been ablated. The deep rectangle scleral lake was then enlarged to about 5 × 4 mm^2^ with a crescent blade. A 1 × 1 mm^2^ sclerostomy was created until the choroid was exposed in each sclerectomy bed with a 15 degree stab blade. The procedures above were repeated in the other three quadrants. The conjunctiva was adequately secured with 8–0 absorbable sutures. The eye was patched with tobramycin-dexamethasone ointment after subconjunctival injection of 5 mg dexamethasone. The surgical procedures are demonstrated in Figure 1 and Supplemental Digital Contents (see Supplementary Video 1).

## Results

The surgery was done in three eyes of three patients (Table 1), who did not respond well to medications. The mean follow-up period was 410 days (range, 34–825 days). Case 1 was followed for 825 days, and Case 3 for 370 days, both demonstrating stable resolution of retinal detachment without recurrence or late complications. Case 2 was followed for 34 days due to geographical reasons but showed complete early resolution.

**Table 1:** Distribution of demographic and clinical variables.

Case	Age	Gender	Laterality	Classification	Axil length/mm	LogMAR BCVA		Follow-up (days)	Macular thickness		Choroidal thickness	
						Preop	Final		Preop	Final	Preop	Final
1	41	F	OS	Type 2	21.84	0.70	0.22	825	181	177	808	623
2	46	M	OS	Type 2	22.84	1.00	0.25	34	235	148	494	396
3	75	M	OD	Type 3	23.60	0.92	0.82	370	NA	NA	NA	NA

LogMAR, logarithm of minimal angle of resolution; BCVA, best-corrected visual acuity.

**Figure 2:**
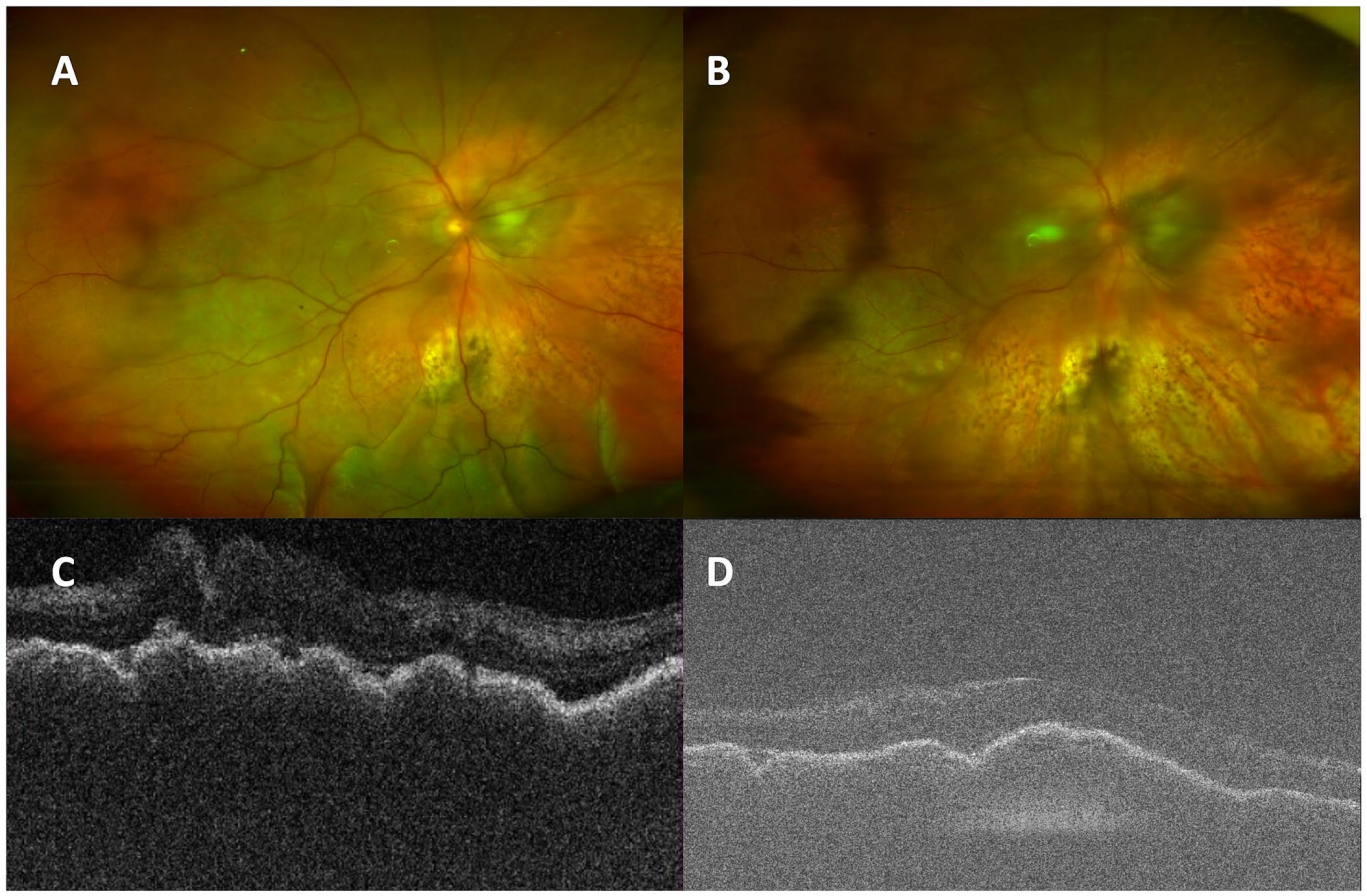
**(A)** Ultrasound biomicroscopy (UBM) demonstrated separation of longitudinal fibers of ciliary muscle with severe ciliary edema in the left eye of Case 2. **(B)** Pre-operative optical coherence tomography (OCT) showed subretinal fluid and thickened choroid. The uveal effusion significantly improved after the surgery as shown by UBM **(C)** and OCT **(D)**.

**Figure 3:**
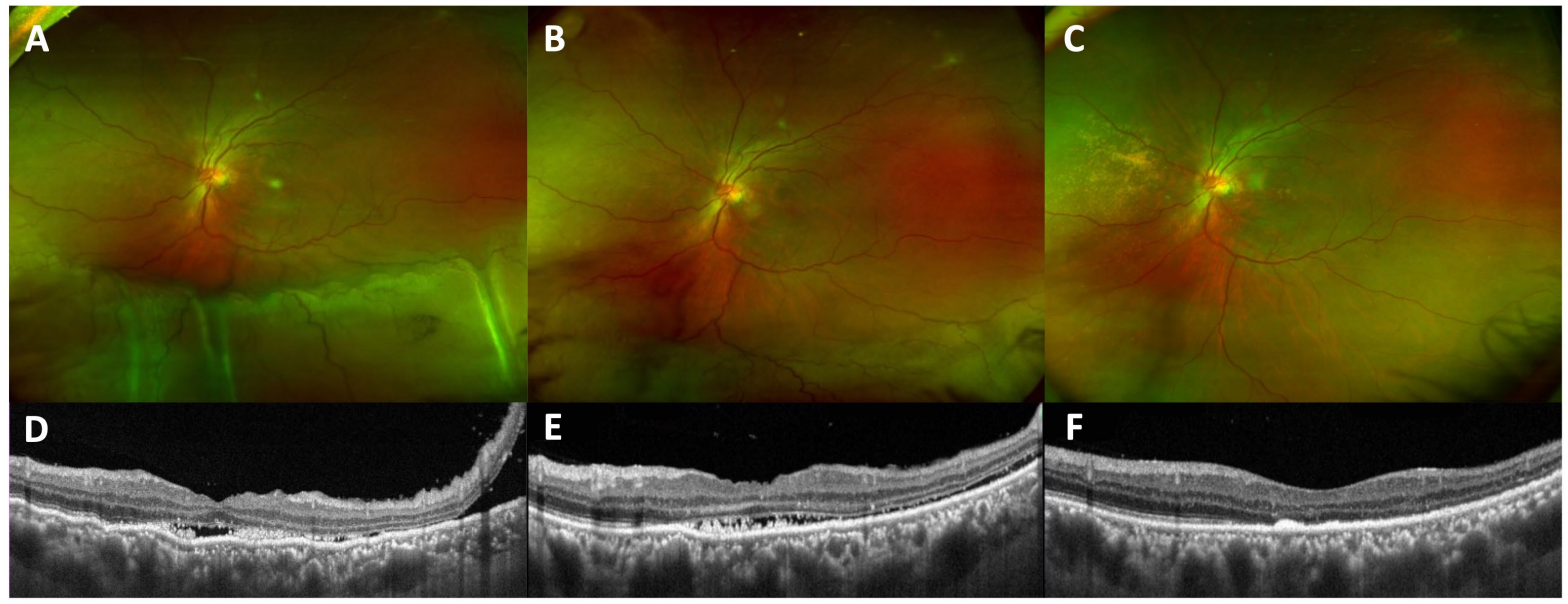
**(A)** Before surgery, there was inferior serous retinal detachment of the left eye. **(D)** Optical coherence tomography (OCT) showed subretinal fluid and thickened choroid. The retina gradually flattened **(B,C)** with absorption of subretinal fluid **(E,F)**.

**Figure 4:**
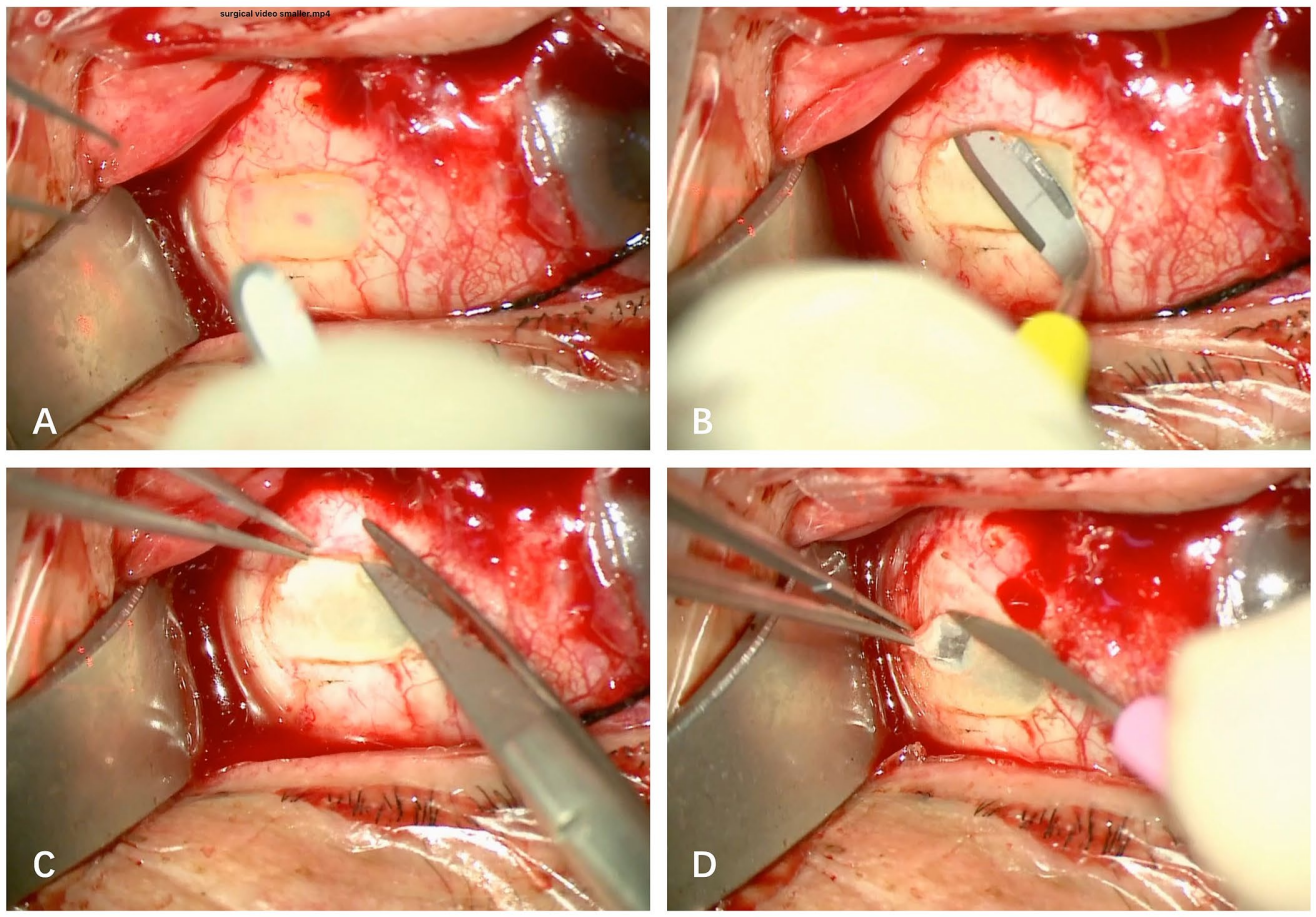
**(A)** Before surgery, the inferior retinal detachment was obvious in scanning laser ophthalmoscopy (SLO). **(C)** Optical coherence tomography (OCT) depicted clear subretinal fluid and macular folds, which greatly improved after surgery **(B,D)**.

For Case 1 (Figure 2), the macular thickness and choroidal thickness decreased from 181 and 808 μm to 177 and 623 μm, respectively, and case 2 (Figure 3), from 235 and 494 μm to 148 and 396 μm, with complete resolution of subretinal fluid. In Case 3, an obvious improvement of retinal detachment and absorption of subretinal fluid was noticed, as shown in Figure 4.

The mean logMAR BCVA improved from 0.87 (Snellen 20/150) preoperatively to 0.43 (approximately Snellen 20/50) postoperatively, and the mean IOP decreased from 19.1 mmHg to 15.8 mmHg at final follow-up. Individual patient trajectories are detailed in Table 1. All patients exhibited no or mild postoperative inflammation, which was well-controlled with local anti-inflammatory eye drops.

## Discussion

Surgical treatment of UES is challenging. Scleral-thinning procedures, mostly partial- or full-thickness sclerectomies, are more commonly used compared to decompression of the vortex veins, which bear a high risk of vortex vein amputation and massive hemorrhage (1). These procedures, however, can also be complicated with scleral ectasia or iatrogenic hemorrhage if the scleral pool is too large or deep. Alternatively, a small or shallow scleral pool would compromise the efficacy to drain the effusion. These considerations may put surgeons in a dilemma to choose from efficacy and safety, limiting the application of sclerectomies in patients with poor response to medications.

In this circumstance, CLASS makes a perfect choice, as the CO_2_ laser ablates only a thin layer of sclera after each application of laser beam, leaving the surgeon enough space to observe the ablated and remnant thickness of sclera and decide if the size and depth of sclera pool is appropriate (7). In our case series, we used CO_2_ laser to make a deep scleral pool semiquantitatively until a bluish hue was observed, which was about 4/5 scleral thickness. The sclera pool was then enlarged to about 5 × 4 mm^2^ followed by a 1 × 1 mm^2^ sclerostomy in three eyes, whose uveal effusion significantly improved after the surgery. The number of laser applications (9–17 times) provides a rough guide, the final endpoint is primarily determined by visualization of the target depth. Treatment of these cases demonstrates the clinical applicability of CLASS with sclerostomy in UES. While we observed a reduction in choroidal thickness in two cases, the exact mechanism of fluid resolution remains speculative. It is unclear whether the benefit is primarily due to direct mechanical drainage through the sclerostomy, enhanced transscleral outflow, or a secondary effect from reduced choroidal congestion.

During the surgery, CO_2_ laser was applied 9–17 times, depending on the choroidal thickness. The parameters used to create the first scleral pool served as good reference for the three other quadrants, which greatly improved the efficiency of the surgery.

In terms of safety, CO_2_ laser ablation of sclera decreases the scleral resistance and improve the uveal-scleral outflow gradually, which theoretically decreases the risk of expulsive suprachoroidal hemorrhage. In addition, leaving a thin layer of sclera may help prevent long-term complications such as scleral ectasia, although extended follow-up in larger cohorts is needed to confirm this. Since the CO_2_ laser may theoretically ablate the outer wall of the choroidal vessels, the sclerostomy was performed manually to avoid choroidal or retinal injuries and expulsive hemorrhage.

The major limitations of the study are the small number of cases and the lack of a control group. Although the follow-up period has been extended for two patients (825 and 370 days), the overall sample remains too small to draw definitive conclusions about long-term efficacy or rare complications. In addition, there was no patient with type 1 UES in our case series, and the applicability of this technique to type 1 UES remains to be determined. One patient with type 3 UES was included. This patient was offered surgery due to persistent, vision-threatening exudative retinal detachment despite maximal medical therapy, after a thorough discussion of the potentially guarded prognosis associated with this subtype. This case showed anatomical improvement but limited visual gain, likely due to chronic macular changes associated with prolonged detachment. Future studies may address these limitations by working in joint effort among groups and observing the long-term efficacy and safety of this novel surgical technique.

In conclusion, CLASS combined with sclerostomy is a semiquantitative and safe surgical technique for managing UES patients who do not respond to medication. Extended follow-up in two cases (up to 2.3 years) suggests that the initial resolution of retinal detachment can be maintained without late complications. However, larger prospective studies are needed to confirm these findings and establish long-term efficacy and safety.

## Data Availability

The original contributions presented in the study are included in the article/Supplementary material, further inquiries can be directed to the corresponding author.
